# The regulatory approval of anamorelin for treatment of cachexia in patients with non‐small cell lung cancer, gastric cancer, pancreatic cancer, and colorectal cancer in Japan: facts and numbers.

**DOI:** 10.1002/jcsm.12675

**Published:** 2020-12-31

**Authors:** Hidetaka Wakabayashi, Hidenori Arai, Akio Inui

**Affiliations:** ^1^ Department of Rehabilitation Medicine Tokyo Women's Medical University Hospital 8‐1, Kawada‐cho, Shinjuku‐ku Tokyo Japan; ^2^ National Center for Geriatrics and Gerontology Obu Japan; ^3^ Pharmacological Department of Herbal Medicine Kagoshima University Graduate School of Medical & Dental Sciences Kagoshima Japan

## Abstract

Anamorelin is a ghrelin receptor agonist that can be administered orally and thought to improve cancer cachexia by improving appetite and increasing serum insulin‐like growth factor‐1. Anamorelin was not approved for use in Europe. In contrast, the use of anamorelin for cancer cachexia in four types of cancer (non‐small cell lung cancer, gastric cancer, pancreatic cancer, and colorectal cancer) was approved in Japan on 11 December 2020. Phase 2 trial (ONO‐7643‐04) for the treatment of patients with non‐small cell lung cancer and cachexia resulted in 1.56 kg lean body mass increase assessed by dual‐energy X‐ray absorptiometry (DXA). Another study for advanced and unresectable gastrointestinal (colorectal, gastric, or pancreatic) cancer showed 1.89 ± 0.36 kg improvement in lean body mass. Skeletal lean body mass assessed by DXA is important for diagnosing sarcopenia and cachexia in Asia. The approval of anamorelin is expected to change clinical practice of cancer cachexia in Japan and hopefully in other countries. In the past, cachexia was rarely diagnosed in Japan, because it was often thought that cachexia meant terminal stage. The dissemination of clinical findings on anamorelin from Japan, as well as the creation of consensus papers and clinical practice guidelines for cachexia in Japan and Asia, will be required to promote international expansion in the future.

Pharmacotherapy for cachexia is limited. The Food and Drug Administration approved human growth hormone for people with acquired immunodeficiency syndrome‐related cachexia more than 20 years ago in the USA.^1^ Megestrol acetate can be used for anorexia and cachexia in patients with cancer or acquired immunodeficiency syndrome in Korea. However, growth hormone and megestrol acetate were not approved in Japan for cachexia. The American Society of Clinical Oncology guidelines for cancer cachexia published in 2020 recommend drug intervention only by progesterone analogues and corticosteroids for appetite improvement and weight gain.^2^ Corticosteroids are recommended only in a short term at a dose of 3–4 mg of dexamethasone per day. However, other drugs including anamorelin are not included as a treatment option in the current recommendations.^2^

Anamorelin is a ghrelin receptor agonist that can be administered orally.^3^ Ghrelin is a peptide secreted mainly by the stomach. It is an endogenous agonist of the growth hormone receptor type 1a, which regulates energy metabolism *in vivo* by promoting growth hormone secretion, increasing appetite, and promoting lipogenesis.^4^ Anamorelin is a selective oral ghrelin‐like agonist that acts on ghrelin receptors in the stomach. Anamorelin is thought to improve cancer cachexia by improving appetite and increasing serum insulin‐like growth factor‐1.^5^, ^6^

Anamorelin was not approved for use in Europe. The Committee for Medicinal Products for Human Use concluded that the ROMANA studies^7^, ^8^ showed a marginal effect of Adlumiz (anamorelin) on lean body mass and no proven effect on hand grip strength or patients' quality of life in 2017.^9^ Moreover, the safety data of the drug had not been recorded adequately.^9^

In Japan, the Pharmaceuticals and Medical Devices Agency is a regulatory agency, working together with the Ministry of Health, Labor and Welfare for drug approval. They conduct scientific reviews of application of medical devices as well as pharmaceuticals and are responsible for monitoring their post‐marketing safety. They set a target for the total review time for new drugs at a median of 12 months for standard review products.

The use of anamorelin for cancer cachexia was approved in Japan for the first time in the world on 11 December 2020. An application was filed in Japan for manufacturing and marketing approval of anamorelin with the planned indication of ‘improvement of weight loss and anorexia in cancer cachexia’ in November 2018. The two clinical studies on which the application was based are as follows. Phase 2 trial (ONO‐7643‐04) for the treatment of patients with non‐small cell lung cancer and cachexia resulted in 1.56 kg lean body mass increase assessed by dual‐energy X‐ray absorptiometry (DXA).^5^ Changes in lean body mass, body weight, and anorexia symptoms showed significant differences in the treatment arm compared with the placebo arm; however, handgrip strength or 6‐min walk test did not show significant differences.^5^ Another study for advanced and unresectable gastrointestinal (colorectal, gastric, or pancreatic) cancer showed 1.89 ± 0.36 kg improvement in lean body mass assessed by DXA.^6^ However, functional endpoints such as muscle strength and physical function were not evaluated in this study.^6^ Most studies showed a significant increase in lean body mass assessed by DXA (*Table* 1).^10^

**Table 1:** Effects of anamorelin on lean body mass

	Anamorelin		Placebo		*P* value
	Mean (kg)	SD (kg)	Mean (kg)	SD (kg)	
Katakami *et al*.^4^	1.38	0.18	−0.17	0.17	<0.001
Hamauchi *et al*.^6^	1.89	0.36			
Takayama *et al*.^10^	1.15	0.31	0.55	0.29	0.0516
	**Median (kg)**	**95% CI (kg)**	**Median (kg)**	**95% CI (kg)**	
ROMANA 1^7^	0.99	0.61 to 1.36	−0.47	−1.00 to 0.21	<0.001
ROMANA 2^7^	0.65	0.38 to 0.91	−0.98	−1.49 to −0.41	<0.001

It was deliberated by the Ministry of Health, Labor and Welfare, Pharmaceutical Affairs and Food Sanitation Council, First Committee on Drugs, and decided to continue deliberation on August 2019. The reason was that there were doubts about the efficacy, safety, and the way information should be provided to medical institutions and patients.

In December 2020, the second round of deliberation was held, and the approval was granted for the indication of cancer cachexia in four types of cancer: non‐small cell lung cancer, gastric cancer, pancreatic cancer, and colorectal cancer.^11^, ^12^ Anamorelin treatment increased lean body mass assessed by DXA and body weight.^5^, ^6^ Assessment of lean body mass by DXA is not recommended for sarcopenia diagnosis in the Sarcopenia Definition and Outcomes Consortium position statements^13^ but is included in the Asian Working Group for Sarcopenia 2019 consensus.^14^ Skeletal lean body mass assessed by DXA is important for diagnosing sarcopenia and cachexia in Asia. It is expected to be officially approved by the end of 2020 at the earliest and by the end of January 2021 at the latest.

Specific dosage and administration, precautions, warnings, contraindications, and post‐marketing surveillance are planned as follows.^11^ The usual adult dosage of anamorelin hydrochloride is 100 mg orally once daily on an empty stomach. However, there are several precautions. If no effect is observed after 3 weeks from the start of administration, administration should be discontinued. There is no experience of administration for longer than 12 weeks. Moreover, there are several warnings. Anamorelin should be used under the supervision of a physician with sufficient knowledge and experience in cancer cachexia. The risks and benefits of administering the drug should be fully explained and made sure that the patient or family understands before administering the drug. As an example of the risks associated with the drug, cardiac risks such as QT interval prolongation may occur. In addition, the drug is contraindicated in patients with a history of hypersensitivity to any of its ingredients or in patients taking concomitant medications. Post‐marketing surveillance is mandatory. The re‐examination period is 8 years.

The approval of the use of anamorelin is expected to change clinical practice of cancer cachexia in Japan and hopefully in other countries. In the past, cachexia was rarely diagnosed in Japan, because it was often thought that cachexia meant terminal stage. Only 17.4% of health care professionals answered that they were evaluated for cachexia.^15^ However, for cachexia due to non‐small cell lung cancer, gastric cancer, pancreatic cancer, and colorectal cancer, cachexia is more likely to be diagnosed and anamorelin used when weight loss of 5% or more is observed in 6 months. Furthermore, it is necessary to increase the awareness of cancer cachexia and to study whether the combination of drug therapy with nutrition therapy,^16^ exercise therapy, and rehabilitation^17^ can improve skeletal muscle function and prognosis in early‐stage patients who meet the criteria for cachexia or even pre‐cachexia. Moreover, it is not realistic to conduct clinical trials for all carcinomas, and the effectiveness of the drug should be verified using common biomarkers.

For future international expansion, it is important to show improvement in muscle strength, physical function, patient‐reported outcomes, and quality of life, as well as appetite and weight, by treating cachexia from multimodal interventions^18^ including anamorelin rather than anamorelin alone. In Asia, such as Korea and Taiwan, anamorelin may be approved for improvement of weight loss and anorexia in cancer cachexia. On the other hand, in Europe and the USA, approval is difficult to obtain unless muscle strength, physical function, and prognosis are improved.^19^ The dissemination of clinical findings on anamorelin from Japan, as well as the creation of consensus papers and clinical practice guidelines for cachexia in Japan and Asia, will be required to promote international expansion.

## Conflict of interest

Hidetaka Wakabayashi, Hidenori Arai, and Akio Inui have declared no conflicts of interest.
